# A human centred innovative approach based on persona in hereditary angioedema

**DOI:** 10.1186/s13023-024-03302-x

**Published:** 2024-08-10

**Authors:** Francesca Perego, Lorenza Chiara Zingale, Azzurra Cesoni Marcelli, Luca Ranucci, Lorenzo Rimoldi, Nurgul Nsanbayeva, Maria Rosaria Natale, Laura Adelaide Dalla Vecchia, Alessandra Gorini

**Affiliations:** 1 HAE Unit, IRCCS Istituti Clinici Scientifici Maugeri, Via Camaldoli 64, 20138 Milan, Italy; 2 Your Business Partner, London, UK; 3 Your Business Partner, Milan, Italy; 4 Dipartimento di Scienze Cliniche e di Comunità, Dipartimento di Eccellenza 2023-2027, Università degli Studi di Milan, Milan, Italy

**Keywords:** Hereditary angioedema, Personas, Human center design, Unmet needs, Prevention, Rehabilitation, Rare disease, Caregiver, Qualitative interviews, Personalisation, Psycholosocial well-being

## Abstract

**Background:**

Hereditary Angioedema (HAE) due to C1-inhibitor deficiency (C1INH) is a rare condition, clinically characterised by recurrent swelling. The unpredictability of attacks affects the patients’ quality of life (QoL). HAE patients and their families have vast unmet physical, psychological, and social needs. A human-centred design (HCD) approach to describing the needs of different user types is to utilise personas, a data-driven narrative tool for communicating user archetypes that capture the individuals’ attitudes, goals, and behaviours. The aim of this study was to create and analyse personas based on HAE patients’ and their caregivers’ interviews. Semi-structured interviews were conducted through anthropological conversations with patients, patient-caregivers (double role of patient and caregiver), and non-affected caregivers. Qualitative and quantitative insights from analyses formed the basis to create personas.

**Results:**

We enrolled 17 subjects: 15 patients (6 of them were patient-caregivers) and 2 non-affected caregivers. The mean age of participants was 50.3 ± 14.4 years. Eight patients were on treatment with prophylactic therapy. The mean percentage score of Angioedema Quality of Life (AE-QoL) for HAE patients was 19.8 ± 12.0. Six personas were identified describing the participants’ personal history, disease management, and needs: four personas referred to patients, one to patient-caregivers, and one non-affected caregiver personas were identified. Across patient personas, the most expressed needs were psychological support and better awareness amongst healthcare professionals. Caregivers, on their side, desired better information about the disease, including the latest therapies, and higher awareness within the community.

**Conclusion:**

A Human Centred Innovative Approach Based on Persona extends beyond the physical symptoms to encompass the psychological and social aspects of the individual's well-being also including the family in the evaluation.

## Background

Hereditary Angioedema (HAE) is a rare inherited and potentially life-threatening condition [1, 2] characterized by intermittent, localized, and self-limiting episodes of swelling in the subcutaneous tissues, resulting from a temporary increase in vascular permeability [3]. In a recent consensus [4], the authors categorized Angioedema (AE) into five primary types and endotypes. HAE is subdivided into HAE-C1INH-type 1 (deficiency of C1INH proteins), HAE-C1INH-type 2 (disfunction of C1INH proteins) and HAE with normal levels of C1INH [5, 6]. The latter is associated with various genetic mutations such as those in factor XII, plasminogen and other related genes [7].The most common form of HAE are C1INH-type 1 and C1INH-type 2. Most patients with HAE report a familial history of the illness in parents or other first-degree relatives [1]. The prevalence of HAE worldwide ranges from 1:10,000 to 1:150,000 and on average is considered to be 1:50,000 [8–10]. In Italy, its prevalence rate is slightly lower with 1 in 64,000 people being diagnosed with HAE. However, statistics show that this number is slowly increasing towards the world average [11].

Symptomatic episodes of HAE are characterized by severe swellings (angioedema) of the mucous membrane in the patients’ arms, legs or face, as well as gastrointestinal attacks [12, 13]. The attack develops gradually over several hours, increases slowly for 12–36 h, and lasts for around two to five days [14]. Most patients do not require admission to the hospital [15]; however, in severe cases, the abdominal lining and/or lining of the upper respiratory tract may also be affected leading to more serious manifestations that in extreme cases could prove fatal [16].

Throughout their lives, HAE patients and their caregivers face numerous disease-specific challenges, influencing the patients’ physical and mental health and limiting them in their everyday routine [17, 18]. The diagnostic process is also complex and prolonged. Since symptoms present in HAE patients are similar to other conditions, such as allergic reactions, gastroenteritis etc., a high number of patients are misdiagnosed and experience delays in getting the right treatment. Studies have reported that the mean delay in diagnosis of HAE-C1INH patients is on average 8.3–10.3 years in Europe [19, 20]. In the process of visiting various physicians, 65% of participants received a misdiagnosis of their condition, with the most common misdiagnoses being allergy/allergic reaction (38%) and appendicitis (17%). In addition, 24% of participants in Europe underwent an unnecessary surgical procedure [20].

The unpredictability of attacks limits the ability of patients to perform daily tasks. In a study involving 92 Hungarian HAE patients over 7 years, the most common attack triggers were physical exertion (71%), mental stress (59%), and mechanical trauma (59%) [21]. Recently, an increased vagal modulation to the sinus node, reflecting localized vasodilation mediated by the release of bradykinin has been identified as an early marker of an impending angioedema attack [22], helping to identify patients at higher risk of attack recurrence. Depression and anxiety are also common in patients with HAE [23]. In a study conducted in the US with 457 HAE patients, 42.5% experienced depression and anxiety symptoms, which may be related to the severity of the chronic disease, to the associated pathophysiologic characteristics, or both [24]. In general, patients who suffer more frequent and severe attacks experience more hindrances to their education and ability to work, with a consequent significant economic burden [25].These data suggest the need for a Human-Centred Design (HCD) approach that focuses on the discovery of users’ unexpressed and unarticulated needs to enable the development of tailored solutions [26]. Such kind of approach includes not only clinical treatment, but also addresses psychosocial issue to lead to prevention of the attacks and improved quality of life [27, 28]. A common human–computer interaction technique utilised by the HCD approach to describe the needs of different user types is called *persona*. Persona is a data-driven narrative tool based on qualitative and quantitative data collected through ethnographic interviews. Personas are composite archetypes derived from this tool that are used during the design process to capture attitudes, goals, and behaviours of real people, to build empathy towards users, and to communicate their needs [29–31]. In health domains, personas are used in the design and human interaction for health technology research; however, personas could be extended to studies of the psychosocial and care experience [32].

Based on this framework the main aim of the present study was to create personas starting from anthropological conversations on HAE C1INH-type 1 and C1INH-type 2 patients and their caregivers. This approach will represent the first attempt to better understand the unmet and unarticulated needs relating to psychosocial aspects and care experiences of this rare disease [33].

## Materials and methods

### Participants

The study included adult female and male C1INH-type 1 and C1INH-type 2 patients, patients who are also caregivers (patient-caregivers) and non-affected caregivers of adult and paediatric HAE patients.

All patients were treated at the HAE Centre of IRCCS Istituti Clinici Scientifici Maugeri in Milan, Italy. Adult patients were approached during their routine clinic appointment by their HAE specialist.

Caregivers were suggested by patient participants or by the physician. The only exclusion criterion adopted for both patients and caregivers was the incapacity to give informed consent.

The study was approved by the ethical committee of the IRCCS Istituti Clinici Scientifici Maugeri (“Copernico” project EC 2651; 29 June 2022). Upon agreement, participants were contacted by a research team member and signed a written informed consent.

### Methods

To describe the lived experiences, personal knowledge and perceptions of HAE patients and their caregivers, the study used the phenomenological method [34, 35]. This approach was chosen because it provides awareness and sensitivity in understanding individuals’ everyday experiences living and managing a chronic rare disease like HAE beyond the traditional quantitative approach to measure health related quality of life (HR-QoL) and disease related Quality of Life (AE-QoL) [36, 37].

The research team was composed by a project manager, a medical anthropologist, a business analyst, and a human-centred designer. After a workshop with the reference clinicians, the team engaged each participant to the study in one-to-one semi-structured interviews, which are referred to as *anthropological conversations*, in order to collect the perspectives of patients and caregivers. The interviews were conducted using the HCD approach [38] focusing on the discovery and deep understanding of users’ needs to enable the development of tailored solutions [26]. In particular, interviews assessed the individual experience with the care provided, living with HAE, as well as unmet and unarticulated needs and desires, and were conducted according to an interview guide including the following five macro-areas of HAE patient’s experience:

1. Personal story and demographics;
2. Clinical History and experience with HAE focusing on psychosocial aspects;
3. Experience with HAE clinics (other clinics and Maugeri HAE Unit);
4. Impact on personal/ psychosocial life;
5. Sources of information about the disease and involvement in HAE community/ patient association/etc.

The contents of the 5 areas are detailed in Table 1.

**Table 1:** Macro-areas of the interviews

Person identification and demographics	Clinical characteristics	Experience with the Centre	Impacts on personal and social life	Access to information and community
Age Gender Ethnicity Living Status Children Highest level of education Work status Residence Type of residence	Diagnosis Age of first symptoms Age of diagnosis Disease activity Other family members affected Number of treated attacks Emergency room visits Pharmacological treatment Side effects of pharmacological treatment Non-pharmacological treatment Surgeries Relationship with GP Psychological treatment Costs	Care pathway before the Centre How/ Why patient arrived to the Centre Waiting time First visit Subsequent/ usual visits Frequency of visits/ check-ups Appointment booking method Treatment/ activities in the Centre Relationship with personnel of the Centre Transportation mode to arrive to the Centre Distance to the Centre Areas of improvement	Physical activity Hobbies, interests Relationship with family Relationship with friends, acquaintances Physical restrictions due to medical condition Emotional impact of medical condition Social impact of medical condition Restrictions at the workplace/ school Support in the workplace/ school	Main source of information about the condition Involvement with a patients’ association Participation in HAE online forums/ social media Involvement with other HAE communities

The interviews were conducted online via a video-telephone software program. Participants could decide whether to give consent to recordings or not.

Data obtained from the interviews were then used to create *Personas*.

Finally, the quality of life was evaluated using a specific questionnaire administered by an anthropologist, the AE-QoL [36]. This questionnaire, which is routinely used in clinical practice, consists of 17 items and assesses impairment in quality of life across four main domains: Functioning, Fatigue/Mood, Fears/Shame, and Food.

Higher scores indicate greater impairment in quality of life.

### Data analysis

#### Coding

All the conversations were conducted in Italian, transcribed verbatim and anonymised. A thematic analysis was used to guide the analysis of data from the anthropological conversations using MAXQDA 2022 software [39] for text analysis and mixed-methods research. The coding scheme was devised based on the five macro-areas defined above. As an iterative process, new codes were added and some were removed/ merged throughout the coding process (Table 2).

**Table 2:** Example of a coding scheme

Codes based on the literature review and defined in the interview guide	Codes added throughout the process:
Code: Management of HAE Sub-codes: Frequency of attacks Description of an attack Attack management Self-administration training Emergency room experience Attack triggers Diary	Code: Management of HAE Sub-codes: Family history Code: Experience with paediatric care Sub-code: Paediatric care experience at the Centre(s) Impact on child’s daily endeavours (e.g. school, nursery)

Three research team members coded the transcripts following the same coding scheme, and afterwards a lead researcher (a medical anthropologist) reviewed all the coded transcripts.

### Personas creation

The research team followed a guideline by Adler [31] on creation of personas based on qualitative data, as well as Ku and Lupton [38] on application of design thinking and human-centred methods in healthcare. A human-centred designer team used the affinity diagram process to group and identify preliminary proto-personas, a working draft of potential participant groups [38]. The affinity diagram process is a design tool used to make sense of large volumes of information by writing down all the relevant notes on post-its and organising them to identify behavioural variables, relevant themes, and emerging insights. Those preliminary personas were used to create document sets, a grouping of documents (transcripts) in MAXQDA 2022, and consequently, display frequency of thematic codes per set. It might happen that the same participant displayed characteristics of several emerging personas. In this case, the transcripts and coded segments were continually checked and discussed with other research team members to confirm the relative position of a participant [26, 29–31, 38].

It is important that personas look real and believable. Therefore, emerging personas were given demographic characteristics based on the quantitative data of the participant samples [31]. Names and photos, on the other hand, are fictional. Each persona’s description includes the personal history, experience within the Centre, HAE management, and needs substantiated by the real quotes. The personas were described in an empathic manner, focusing on the psychosocial aspect of their past and current care experiences.

## Results

### Participant sample characteristics

The cohort consisted of 17 participants: 9 patients (4 female), 6 patient-caregivers (4 female), and 2 non-affected caregivers (1 female). Out of the 15 patients and patient-caregivers 12 were on long term prophylaxis. The mean age of participants was 50.3 ± 14.4 years, 8 patients were on prophylactic therapy, 12 were diagnosed with type 1 (HAE-C1INH-Type1), while 3 were diagnosed with type 2 (HAE-C1INH-Type2). The age at first symptom was 10.4 ± 4.1 years, the delay from the first symptom to diagnosis was 14.6 ± 12.1 years. The mean percentage score of AE-QoL for HAE patients was 19.8 ± 12.0.

Eight patients and 1 caregiver had children. Due to the inherited nature of the condition, some enrolled patients were related (i.e. mother-daughter, first-degree cousins). Regarding the participants’ working status, 10 patients and 2 caregivers had a full-time job, 1 patient had a part-time job, 3 were retired and 1 was unemployed. All participants were Italian.

### Personas

Based on 17 conversations, a total of 6 personas—4 patients, 1 patient-caregiver, and 1 non-affected caregiver personas—were identified. Some of the participants’ quotes from the anthropological conversations are reported below in the description of personas.

### Patient personas

Three out of 4 patient personas emerged based on the severity of the condition (severe, moderate, mild), diagnosis delay and how the condition affects their quality of life and lived experience.

**Fig. 1:**
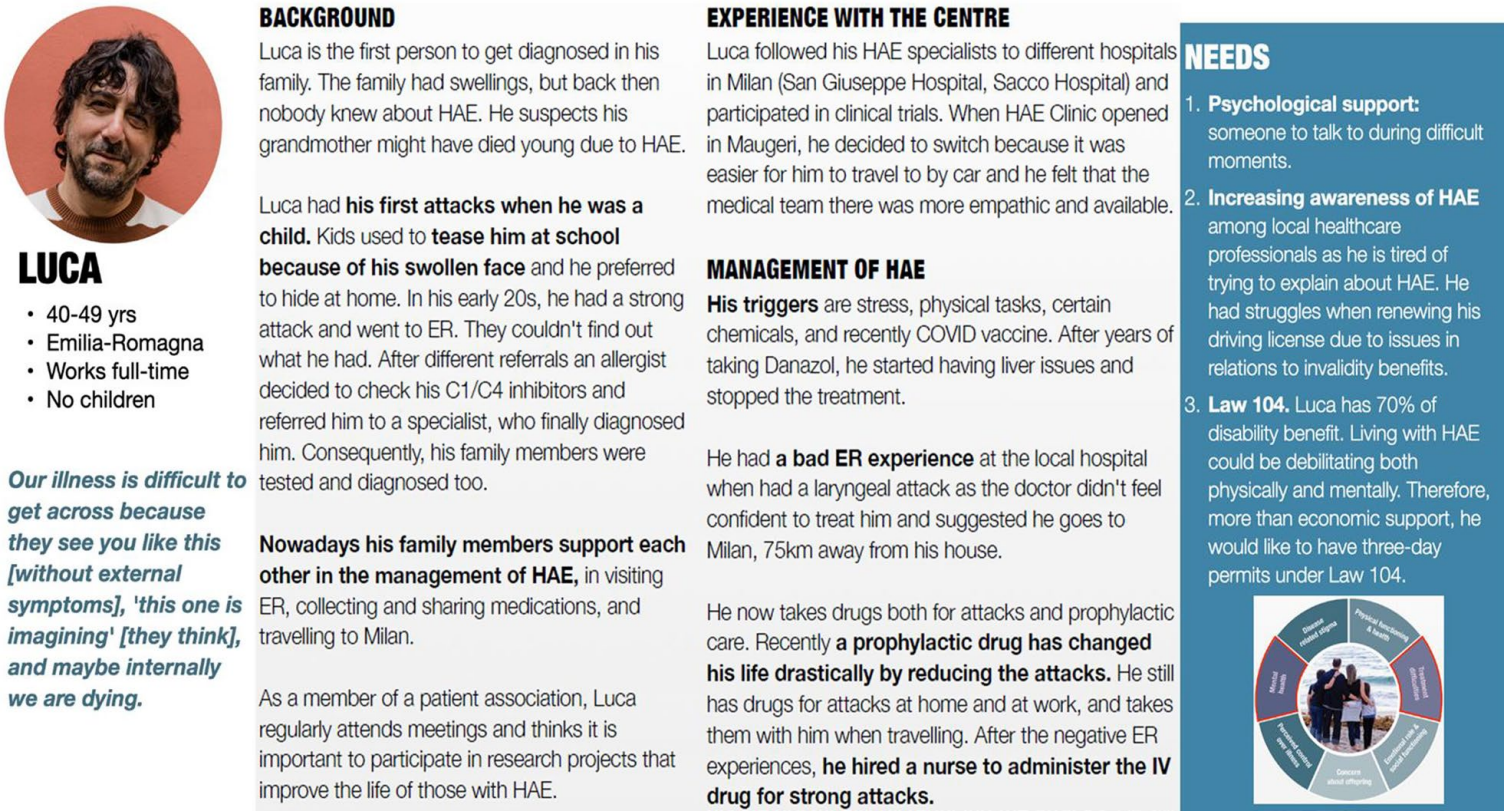
Example of a full persona

The two most represented user groups are Persona 1 (Luca) with severe attacks (n = 5) and Persona 3 (Antonio) with moderate-mild attacks (n = 6) and an explicit family history of HAE. Figure 1 shows an example of full Persona 1—Luca, who represents the group with severe attacks.

Persona 2 (Esther) represents a group of patients (n = 3) who have been recently diagnosed without having an explicit family history. Two out of 15 patients reported strong fear/ terror/ anxiety of attacks before being diagnosed because they did not know the cause of swelling.

*“At the diagnostic stage when I knew there was something wrong but no one understood what it was… it's clear that even trying to find it out had definitely an impact, major diseases like...because clearly if the doctors don't know the symptoms they start from the nastiest, so you start from leukaemia investigation, HIV, cancer and so on...and it is like a roller coaster ride. Last time I was hospitalised I believed until the end that I had bone marrow dysfunction, which means leukaemia...it's tough sorry [cries]”.*

Persona 4 (Mirta) is based on the experience of one elderly person (n = 1) and family stories shared indirectly by other participants (n = 4). Among those who have a family history of HAE (n = 12), four shared a story of elderly family members who experienced physical, psychological, and socio-economic challenges in the past due to undiagnosed/misdiagnosed HAE when little or no information about the condition existed. These past family experiences may have caused distress and trauma to the next generations.

*“My story started with my mom, because I always saw my mom being sick. As long as I can remember, when I was a very small child my mom was always sick, either she was in bed with pains or she had a face that looked like a monster, hands that she couldn't use and nobody understood what was wrong with her. I mean they were very bad and difficult years for a little girl who didn't understand. My poor mom, she also used to go to the doctor but nobody could tell her what was wrong with her, in fact they gave her treatments that did absolutely nothing, or they stuffed her with corticosteroids”.*

### Caregiver personas

Persona 5 (Giusy) represents the experiences of patient-caregivers (n = 6) and patients (n = 4) who have been diagnosed at a paediatric age together with other family members. Among patient-caregivers who are parents some described fear of transmission of the condition to their children, while others expressed hope for the next generations due to new treatment advances.

*“In the beginning, I was terrified of this. If I have to be honest when I found out I was expecting a baby I was hoping it would be a boy, in my head I got the idea... because in my family no one had boys. So, we were only women, so it increased in my head that if it was a boy he wouldn't have it either”.*

Persona 6 (Davide) represents non-affected caregivers (n = 2), as well as experiences of non-affected spouses/ partners indirectly shared by patient participants (n = 2).

*"Having to take care of my loved ones wasn't easy from an emotional point of view: I went through moments of severe stress that literally consumed me.*

*The unpredictability of HAE attacks always makes you worry: you know it's there, that it's latent, that it can arrive at any moment. Now, over the years I have learned to manage anxiety and fear for my family, but I would have liked to have managed the past moments of difficulty better".*

### Participants’ most expressed needs

When closing the conversation, each participant was asked about his/her explicit needs and desires. Unmet and unarticulated needs, on the other hand, were not explicitly voiced but emerged in the conversations organically in relation to participants’ current or past care experiences, challenges and difficulties, including the emotional and psychosocial burden of the condition. During the coding process, those explicit and implicit needs were categorised under the code “Desires”.

Across patient participants and, consequently, among personas created, the most expressed needs were psychological support (Persona 1, Persona 2) and better awareness amongst healthcare professionals (Persona 1, Persona 2, Persona 4).

### Psychological

Five out of 15 patients reported being anxious in the past or at present due to the unpredictability of attacks. Eight patients who were taking the prophylactic treatment mentioned significant improvement in their QoL, also from a psychological point of view, due to having less and milder or nearly no attacks.

All caregivers (6 patient-caregivers, 2 non-affected caregivers) reported the same anxiety for their loved one in the past or present.

Patient and caregivers (n = 5/15) shared a fear of not being listened to by non-HAE healthcare providers due to negative experiences, especially in the Emergency Room (ER).

Three out of 15 participants felt fear and vulnerability during a throat attack because they could not talk and explain their state to a healthcare provider.

It is worth noting that during the interview, among patients, the severity of attacks and the psycho-social impact of the condition were not reported as correlated. Patient participants with a family support network (n = 12/15), which includes other HAE-affected family members, had a better overall understanding of the condition and management of attacks. While patients (n = 3/15) without a clear family history of HAE experienced strong emotional distress before and after receiving a precise diagnosis.

### Knowledge

Eleven patients reported a negative experience in the ER due to a lack of HAE knowledge.

Six patients mentioned a lack of HAE knowledge among general healthcare providers, institutions, and society. Four patients reported difficulties with finding doctors for other health issues (i.e., a dentist) because of HAE and would hide their condition.

Other needs included disability benefits, more information for women about the impact of the condition on fertility and pregnancy, a dedicated phone line for emergencies, new drugs (i.e., that can be taken orally), and bureaucratic challenges between different regions of Italy.

### Caregivers most expressed needs

Patient-caregivers (*n* = 6) and caregivers (*n* = 2) wanted better information about the condition, such as informative newsletters about novel treatments (*n* = 2), HAE cards (*n* = 2), and information for schools/ other facilities that children affected by HAE attend (*n* = 2).

Table 3 gives an extended overview of all the personas with a brief description and their needs.

**Table 3:** Extended summaries of personas (insert the table at the end of the results)

Persona	Demographics	Brief description	Needs
**LUCA**	**Age:** 40–49 years **Work:** full-time **Family status:** single, no children	**Quote:*** It is difficult for people to understand our illness because they see us [without external symptoms], "this one is dreaming" [they think], and maybe internally we are dying* First to be diagnosed in the family due to strong attacks Started the diagnosis chain in his family Severe attacks Prophylactic treatment	Psychological support: someone to talk to during difficult moments Increasing awareness of HAE among local healthcare professionals as he is tired of trying to explain HAE. He had struggles when renewing his driving licence due to issues concerning disability benefits Law 104. Luca has 70% of disability benefits. Living with HAE could be debilitating both physically and mentally. Therefore, more than economic support, he would like to have three-day permits under Law 104
**ESTHER**	**Age:** 30–39 years **Work:** part-time **Family status:** married, with a small child not diagnosed yet	**Quote:** *One of the 800,000 doctors I went to…because I went to all kinds of doctors, I went at one point to the angiologist, to the rheumatologist, to any [specialist], until the last one suggested that I get an immunological examination prescribed* Experienced years of diagnosis delay that left her traumatised Not aware of family members affected Mild to moderate attacks Attack treatment only	Psychological support: years of diagnosis delay traumatised her Increasing awareness of HAE among regional and local healthcare providers (ER, GP, paediatricians, pharmacists, gynaecologists, gastroenterologists, etc.) More information for women about inherited disorders like HAE and pregnancy, and motherhood
**ANTONIO**	**Age:** 50–59 years **Work:** full-time **Family status:** married, with 2 adult children	**Quote:** *I always lived my life, since it's a psychological thing, even if I swelled up I did sports so my body was stressed no matter what. However, occasionally I am terrified of having a throat attack even though it hasn't yet happened* Got diagnosed accidentally as HAE didn't limit his life Family members and children affected by HAE Mild and less frequent attacks Attack treatment only	Email newsletter: information about the latest treatment options, research advances and events, as well as opportunities to take part in studies could be shared with people with HAE
**4. MIRTA**	**Age:** 80–89 years **Work:** retired **Family status:** widow, with 3 adult children	**Quote: ***It was enough to say "I have angioedema" that they didn't do anything to you anymore, they treated you like a plague victim, no one wanted to give you medicine anymore, they didn't know how to do it. I often and willingly denied that I had angioedema* A senior lady who suffered a lot in the past due to HAE, including the trauma of finding her father dead, suffocated due to HAE Has adult children affected by HAE Age-related comorbidities Severe attacks in the past, now mild Attack treatment only	Improve HAE ID cards. ID cards are a great way to let people know in cases of emergency about the condition of the person especially when they cannot talk or breathe List of specialists: a list should be made available with all the local specialists that know about HAE that a person with HAE can go to for other health issues More HAE centres. There is no centre in my region. She would like more specialist centres so that people affected with HAE don’t have to travel far for a visit
**GIUSY**	**Age:** 30–39 years **Work:** full-time **Family status:** married, with one child diagnosed with HAE	Quote: *I try to teach, as much as I can, a 7-year-old girl. I try to do it by calming her down, saying, "Do you see Mommy [how Mommy injects medicine]?"* Diagnosed at paediatric age with her entire family Her child has HAE Moderate attacks Prophylactic treatment	24 h call centre: a dedicated emergency number available 24 h for people to call when needed rather than having to call their specialists at their private mobile number (clinicians may not be available) More defined paediatric care pathway: a more defined care pathway and regular checkups should be put in place, especially for children Increasing awareness among kindergarten and school teachers about rare diseases like HAE (ex. Information brochures)
**DAVIDE**	**Age:** 30–39 years **Work:** full-time **Family status:** married, with one child diagnosed with HAE	Quote: *With [the child] I can define the attack in the sense that I just had to see… one day my dad brought [the child] home he says "I don't know if she has a stomachache". I saw her in the bathroom, I looked at her face and I knew she had an angioedema attack* A non-affected caregiver His wife and daughter are affected by HAE	Information for caregivers and parents about the latest therapies (to be secure about the future of their child) Psychological support. Over the years he learned to manage his anxiety and fear for his family, but he wishes he managed better past difficult moments

## Discussion

In the realm of rare diseases, the emphasis on treating symptoms rather than achieving a complete cure of the affected individual stems from the unique challenges presented by these conditions. Rare diseases, often characterized by their limited prevalence and insufficient understanding, frequently lack early diagnosis and definitive cures due to the complexities of their underlying mechanisms. As a result, the medical approach tends to prioritize managing and alleviating specific symptoms that impact patients' daily lives, aiming to provide relief and improve functionality. The administration of ad hoc questionnaires for the evaluation of the QoL, such as AE-QoL [36] and HAE QoL [40], is routinely used and recommended by guidelines [41]. They are extensively applied as a patient related outcome measure in the evaluation of the efficacy of therapy, whether the on-demand treatment or prophylaxis [42]. However, even with highly effective treatments out of the four domains evaluated by the AE-QoL, the more impactful improvement can be observed inside the “functionality” domain (ref HELP study). In many instances, this approach enhances the overall QoL of affected individuals, but a complete cure may remain elusive.

Furthermore, the individualized nature of rare diseases necessitates a personalized treatment strategy. Tailoring interventions to manage symptoms acknowledges the diversity of manifestations within a particular rare disease and recognizes that each patient may experience a unique set of challenges. This personalized approach extends beyond the physical symptoms to encompass the psychological and social aspects of the individual's well-being.

Creating detailed, fictional representations of typical patients and caregivers, the persona-based approach allowed us to identify a total of 6 personas—4 patients, 1 patient-caregiver, and 1 non-affected caregiver personas—expressing the diverse needs, preferences, and characteristics of individuals who live with a rare and disabling disease, such as HAE.

This approach also brought to the identification of the most expressed patients’ needs including psychological support, better awareness amongst healthcare professionals, disability benefits, more information about the impact of the condition on fertility and pregnancy, a dedicated phone line for emergencies, new drugs (ex. that can be taken orally), and bureaucratic challenges between different regions of Italy. From the side of patient-caregivers and caregivers, better information about the condition, such as informative newsletters about novel treatments, and information for schools and other facilities for children affected by HAE emerged as the primary needs. Of interest, in our interviews, severity of attacks and the psycho-social impact of the condition were not reported as correlated. This is partially in contrast with previous work [43] in which the high attack frequency is a major determinant in the management of home therapy since in the semi-structured interviews there was not a specific question addressing this item.

Obtaining different archetypal individual characters and knowing their often unexpressed, unknown and unmet needs is essential for improving healthcare. It allows healthcare providers and systems to tailor their services to better meet the expectations and requirements of those they serve, enhancing empathy, communication, and the overall quality of care. The creation of archetypal characters is supported by a shared methodology. This is at the same time a scientific and experience-based approach that overcomes the limitation of the single case-report and website reports.

From a more general perspective, the approach used in this study is in line with the well-known Value-Based Healthcare, a model of designing healthcare systems with the rationale that achieving high value for patients must become the overarching goal of healthcare delivery. The value is defined as the health outcomes that matter to patients relative to the cost of achieving these outcomes and should always be defined around the user’s needs, to develop personalised solutions [44]. This concept applies to the entire care pathway, in particular for chronic conditions, including rare diseases, that often require long-time and resource-intensive care management [45].

In the modern approach to care, the long-time care management should include preventive strategies and rehabilitation programs, that refer to a multidisciplinary approach aimed at restoring, maintaining, preventing relapses, and improving the individual's physical, cognitive, and/or psychosocial functions and overall well-being. In the case of HAE patients, who often present significant limitations in their level of autonomy, as well as in private and working-related relationships, rehabilitation, intended as the achievement of the highest possible level of independence and participation in daily life and working activities, becomes a fundamental part of the care process. To be effective in terms of health results and costs, rehabilitation also benefits from the proposed approach focused on personalized interventions centred on the individuals’ needs and values [46, 47].

The study has some limitations. Firstly, pediatric/adolescent subjects were not included in the creation of the personas, despite the fact that HAE often starts at this age and can significantly impact quality of life. Another limitation is the small sample size, as the study involved only 17 participants. This relatively small sample size may affect how generalizable the findings are, as the experiences and needs identified may not fully represent the broader population of individuals with HAE. Additionally, conducting the study at a single center may limit the external validity of the results which may also depend on cultural differences.

In conclusion, we argue that a better understanding of unmet and unarticulated needs, relating to psychosocial aspects and care experiences of patients and their caregivers may improve patient experience, and increase organisational efficiency and cost-effectiveness of HAE care. By using persona as a narrative tool and following an innovative value-based healthcare research, care providers and other stakeholders may be able to make better-informed decisions for the management of people affected by rare diseases and enhance resource utilisation. While the ultimate goal in healthcare remains the discovery of curative interventions, the current reality of rare diseases underscores the importance of a holistic and needs-focused approach.

## Data Availability

Original data are derived from patient’s personal interviews containing sensitive data. Patient-level data can be made available from the corresponding author after discussion with the trial management committee. We provide the data extrapolated by the MAXQDA software. The experimental data and the simulation results that support the findings of this study are available in Figshare with the identifier https://doi.org/10.6084/m9.figshare.25585254.

## Abbreviations

AE-QoL: Angioedema Quality of Life
GP: General Practitioner
HAE: Hereditary Angioedema
HAE-C1-INH: Hereditary Angioedema due to C1-inhibitor deficiency
HCD: Human Centered Design
HR-QoL: Health-Related Quality of Life
